# MicroRNA-122 Triggers Mesenchymal-Epithelial Transition and Suppresses Hepatocellular Carcinoma Cell Motility and Invasion by Targeting RhoA

**DOI:** 10.1371/journal.pone.0101330

**Published:** 2014-07-03

**Authors:** Sheng-Chun Wang, Xiao-Lin Lin, Jing Li, Ting-Ting Zhang, Hui-Yan Wang, Jun-Wen Shi, Sheng Yang, Wen-Tao Zhao, Rao-Ying Xie, Fang Wei, Yu-Juan Qin, Lin Chen, Jie Yang, Kai-Tai Yao, Dong Xiao

**Affiliations:** 1 Cancer Research Institute, Southern Medical University, Guangzhou, China; 2 Institute of Comparative Medicine & Laboratory Animal Center, Southern Medical University, Guangzhou, China; Wayne State University School of Medicine, United States of America

## Abstract

The loss of microRNA-122 (miR-122) expression is strongly associated with increased invasion and metastasis, and poor prognosis of hepatocellular carcinoma (HCC), however, the underlying mechanisms remain poorly understood. In the present study, we observed that miR-122 over-expression in HCC cell lines Sk-hep-1 and Bel-7402 triggered the mesenchymal-epithelial transition (MET), as demonstrated by epithelial-like morphological changes, up-regulated epithelial proteins (E-cadherin, ZO-1, α-catenin, occludin, BVES, and MST4), and down-regulated mesenchymal proteins (vimentin and fibronectin). The over-expression of miRNA-122 also caused cytoskeleton disruption, RhoA/Rock pathway inactivation, enhanced cell adhesion, and suppression of migration and invasion of Sk-hep-1 and Bel-7402 cells, whereas, these effects could be reversed through miR-122 inhibition. Additional studies demonstrated that the inhibition of wild-type RhoA function induced MET and inhibited cell migration and invasion, while RhoA over-expression reversed miR-122-induced MET and inhibition of migration and invasion of HCC cells, suggesting that miR-122 induced MET and suppressed the migration and invasion of HCC cells by targeting RhoA. Moreover, our results demonstrated that HNF4α up-regulated its target gene miR-122 that subsequently induced MET and inhibited cell migration and invasion, whereas miR-122 inhibition reversed these HNF4α-induced phenotypes. These results revealed functional and mechanistic links among the tumor suppressors HNF4α, miR-122, and RhoA in EMT and invasive and metastatic phenotypes of HCC. Taken together, our study provides the first evidence that the HNF4α/miR-122/RhoA axis negatively regulates EMT and the migration and invasion of HCC cells.

## Introduction

Hepatocellular carcinoma (HCC) is one of the most prevalent human malignancies. The elucidation of the molecular mechanisms underlying the tumorigenicity, invasion and metastasis of HCC is critically important for the development of novel treatments for this disease.

MicroRNA-122 (miR-122) is the most abundant microRNA (miRNA) in the liver, accounting for approximately 70% of the total miRNAs in this organ [1]–[5]. Previous studies have demonstrated that miR-122 played multiple roles in the development and differentiation of live cells [1]–[5], liver homeostasis [6], hepatic fatty acid and cholesterol metabolism [2], [6]–[9], hepatic insulin resistance [10], liver fibrosis and cirrhosis [6], [8], [11]–[13], inflammation [6], [8], [9], [11], [14], [15], and modulation of hepatitis C virus (HCV) replication [1]–[5], [14].

The persistent expression of miR-122 has been detected during specialization in the adult liver, and the loss or down-regulation of miR-122 expression has been associated with HCC development and progression [6], [8], [9], [16]–[20]. In recent years, increasing evidence has shown that miR-122 is a tumor suppressor miRNA that negatively regulates cancer cell proliferation [16], [21]–[24], apoptosis [16], [22]–[25], drug resistance [16], [24]–[26], and invasion and metastasis [16], [17], [21], [23], [25], [27], [28].

It has been observed in previous studies that epithelial-like phenotypes were triggered by miR-122 over-expression in hepG2 and Malhlavu cells [16], [25], [28], indicating that miR-122 induces a mesenchymal-epithelial transition (MET) phenotype. For example, miR-122-expressing hepG2 cells exhibited increased E-cadherin expression [25] and decreased vimentin expression [16]. However, it remains unknown how miR-122 induces MET at the molecular level.

The epithelial-mesenchymal transition (EMT), a reverse process of MET, is a critical event in tumor invasion and metastasis that cause the majority of cancer death [29]–[31]. Therefore, understanding the molecular mechanisms of EMT may lead to the development of novel interventions for cancer metastasis.

Given the important roles of miR-122 in HCC invasion and metastasis [16], [17], [21], [23], [25], [27], [28], we aim to investigate the mechanisms of miR-122-induced MET and inhibition of migration and invasion. The candidate transcription factor regulating the expression of miR-122 and the molecular target of miR-122 were also identified and investigated.

## Materials and Methods

### Cell lines and cell culture

The human HCC cell lines, Bel-7402 and Sk-hep-1, were purchased from the Cell Bank of the Chinese Academy of Sciences (Shanghai, China). HEK293T cells were obtained from the American Type Culture Collection (ATCC). The cells were cultured in Dulbecco’s modified Eagle’s medium supplemented with 10% fetal bovine serum (FBS) in a humidified incubator with 5% CO_2_ at 37°C.

### Plasmids

Dr. Eithan Galun (Hadassah University Hospital, Jerusalem, Israel) generously provided the following lentiviral vectors: (1) pSIN18.cPPT.H1p.miR-122.hEF1ap.RFP.WPRE [a lentiviral vector expressing wild-type (WT) hsa-miR-122 (pLV-miR-122)] and (2) pSIN18.cPPT.H1p.hEF1ap.RFP.WPRE (empty vector, pLV-con) [32].

DNA fragments containing coding sequences for HNF4α, RhoA-wt, and RhoA-T19N were amplified from plasmids pCR4-TOPO-HNF4a (Open Biosystems), pcDNA3-EGFP-RhoA-wt (Addgene), and pcDNA3-EGFP-RhoA-T19N (Addgene), respectively. The PCR amplicons were inserted into the multiple cloning sites of lentiviral vector of pHAGE-fullEF1a-MCS-IZsGreen (pLV-GFP as empty vector) (a generous gift from Dr. Jeng-Shin Lee, Harvard Gene Therapy Initiative, Harvard Medical School) to generate lentiviral vectors, pLV-HNF4α, pLV-RhoA-wt, and pLV-RhoA-T19N, respectively. The complete coding sequences for HNF4α, RhoA-wt or RhoA-T19N were confirmed through DNA sequencing.

The lentiviral packaging plasmids psPAX2 and pMD2.G were kindly provided by Dr. Didier Trono (University of Geneva, Geneva, Switzerland). The dual luciferase reporter gene plasmids, pLuc-RhoA-3′-UTR-wt and pLuc-Rac1-3′-UTR-wt, containing the putative miR-122 binding site at the 3′-UTR of RhoA and Rac1 mRNAs, respectively, were purchased from Kangbio (Shenzhen, China).

### Lentivirus production and transduction

Recombinant lentiviruses (LV-con, LV-miR-122, LV-GFP, LV-HNF4α, LV-RhoA-wt, and LV-RhoA-T19N) were generated as previously described [33]. Briefly, to produce virus particles expressing the empty vector or target genes (miR-122, HNF4α, RhoA-wt, and RhoA-T19N), HEK293T cells (maintained in 10% FBS) were transfected with lentiviral vectors and packaging plasmids (psPAX2 and pMD2.G) using Lipofectamine 2000 reagent (Invitrogen) according to the manufacturer’s instructions. The packaged lentiviruses carrying pLV-con, pLV-miR-122, pLV-GFP, pLV-HNF4α, pLV-RhoA-wt, and pLV-RhoA-T19N were named as LV-con (used as a control), LV-miR-122, LV-GFP (used as a control), LV-HNF4α, LV-RhoA-wt, and LV-RhoA-T19N, respectively. At 48 h post-transfection, the supernatant was harvested and subsequently used to infect Bel-7402 and Sk-hep-1 cells.

### miRNAs transient transfection

Human miR-122 mimics, mimic controls, miR-122 inhibitors, and inhibitor controls were purchased from RiboBio Co., Ltd (Guangzhou, China). The miRNAs (100 nM) were transiently transfected into target cells using the Lipofectamine 2000 reagent (Invitrogen) according to the manufacturer’s instructions.

### RNA isolation and quantitative real-time PCR

For mRNA analyses, total RNA from HCC cells was extracted using Trizol Reagent (TaKaRa) according to the protocol provided by the manufacturer. Total RNA was reversely transcribed with the PrimeScript RT reagent Kit (TaKaRa). Quantitative real-time PCR (qRT-PCR) for the expression of mRNA analysis was performed using SYBR Green qRT-PCR master mix (TaKaRa) as described using GAPDH for normalization on a Stratagene Mx3005P qRT-PCR System. The primers used for the amplification of the indicated genes were listed in Table S1 and S2 in File S1. All samples were normalized to internal controls and fold changes were calculated through relative quantification (2^−△△Ct^).

### Western blot analysis and Rho-GTPase activation assay

Protein lysates were separated by SDS-PAGE, and electrophoretically transferred to PVDF (polyvinylidene difluoride) membrane. Then, where relevant, the blots were probed with the primary antibodies as labeled in the corresponding Figs, followed by HRP (horseradish peroxidase)-labeled goat anti-mouse or goat anti-rabbit IgG, and the signals were detected using enhanced chemiluminescence (ECL). β-actin was used as a protein loading control. For origin and description of all antibodies used in this study see Table S3 in File S1.

Rho-GTPase activation assay was carried out according to the methods described previously [34]. In brief, PAK1 PBD-agarose (for isolating Rac1-GTP and cdc42-GTP) and rhotekinagarose (for isolating Rho-GTP) (Millipore) were used to pull down the GTP-bound form of Rho-GTPase according to the manufacturer’s manual. The levels of active Rac1, cdc42, and RhoA were detected by Western blot using specific polyclonal anti-Rac1 (1∶1000), anti-cdc42 (1∶250), and anti-RhoA (1∶375) antibody (Millipore).

### F-actin cytoskeleton staining analysis

F-actin staining was performed according to the manufacturer’s instructions. Fixed cells were incubated with 100 nM working stock of Acti-stain 488 phalloidin (Cytoskeleton). Cells were counterstained with DAPI (Sigma) and imaged with a confocal laser-scanning microscope (Olympus FV1000).

### Transwell migration assay and Boyden invasion assay

For transwell migration assay, 1×10^5^ cells were seeded into the upper chamber (BD Biosciences, MA) with serum-free DMEM. Boyden invasion assay was performed using matrigel (BD Biosciences, MA) in the upper chamber. DMEM with 10% FBS were put into lower compartment as chemo-attractant. Cells were allowed to migrate for 12 hours. Remaining cells in the upper chamber were scraped out by cotton swap. The cells that had migrated to the lower surface of the membrane were fixed with 100% methanol and stained with hematoxylin solution (Sigma), followed by counting in five random optical fields.

### Cell adhesion assay

Cell adhesion experiment was carried out according to the methods described previously [34]. In brief, cells were seeded on culture dishes and grown to ∼90% confluence. The confluent monolayers were then washed with PBS and treated with 0.05 mM EDTA. Cells detachment was examined at different time intervals (2, 10, 20 and 30 min) under a phasecontrast microscope. Cell adhesion was analyzed by counting the adherent cells.

### mRNA microarray analysis

Expression microarray analysis was carried out with commercially available Affymetrix Human Gene U133 Plus 2.0 array according to the Affymetrix standard protocol, which carried 47,000 transcripts representing 38,500 well-characterized human genes. All the hybridization procedures and data analysis were performed by Capital Bio Corp. (Bejing, China). Total RNA samples were isolated from liver cancer cells (Sk-hep-1 cells) using Trizol reagent (Invitrogen). Briefly, total RNA was used to synthesize cDNA in an in vitro transcription reaction, and then cDNA was fluorescently labeled by Cy5 or Cy3-CPT with Klenow enzyme. Labeled cDNA was then hybridized to Affymetrix Human Gene U133 Plus 2.0 arrays. Hybridization was processed at 45°C, with rotation for 16 h (Affymetrix GeneChip Hybridization Oven 640). Chips were then washed and stained in the Affymetrix Fluidics Station 450. Hybridization signals were scanned with a Lux-Scan 3.0 scanner (Capital Bio. Corporation, Beijing, China). The resultant images were digitized with Genepix Pro 6.0 software (Axon Instruments, Foster City, CA, USA).

### Scanning electron microscopy

Scanning electron microscopy was performed according to the methods described previously [34]. Cells were fixed with 1% osmium tetroxide and 2.5% glutaldehyde followed by stepwise ethanol dehydration. After the step of critical point drying, slides were mounted on silver paste. Images were scanned and captured under ×3,000 and ×6,000 magnifications using the quanta 200 scanning electron microscope (Nikon S-3000N). Data were processed with Adobe Photoshop 7.0 software.

### Luciferase reporter assay

The cells were seeded in triplicate into 24-well plates (5×10^4^/well) and cultured for 24 h. HEK293T and Bel-7402 cells were transfected with dual luciferase reporter gene plasmids (pLuc-RhoA-3′-UTR-wt or pLuc-Rac1-3′-UTR-wt (Kangbio, Shenzhen, China) and miR-122 mimics, mimic controls, or miR-122 mimics and miR-122 inhibitors using Lipofectamine 2000 reagent (Invitrogen). Luciferase and Renilla activities were analyzed at 48 h post-transfection using the Dual Luciferase Reporter Assay Kit (Promega) according to the manufacturer’s instructions.

### Statistical analysis

Data were presented as mean±SD unless otherwise indicated of at least 3 independent experiments. Statistical analysis was performed using a SPSS 13.0 software package. Two-tailed Student’s t test was used for comparisons of 2 independent groups. Statistical significance was assessed by the Student’s t-test (*p<0.05; **p<0.01).

## Results

### miR-122 trigged MET and inhibited the motility and invasion of HCC cells

**Figure 1:**
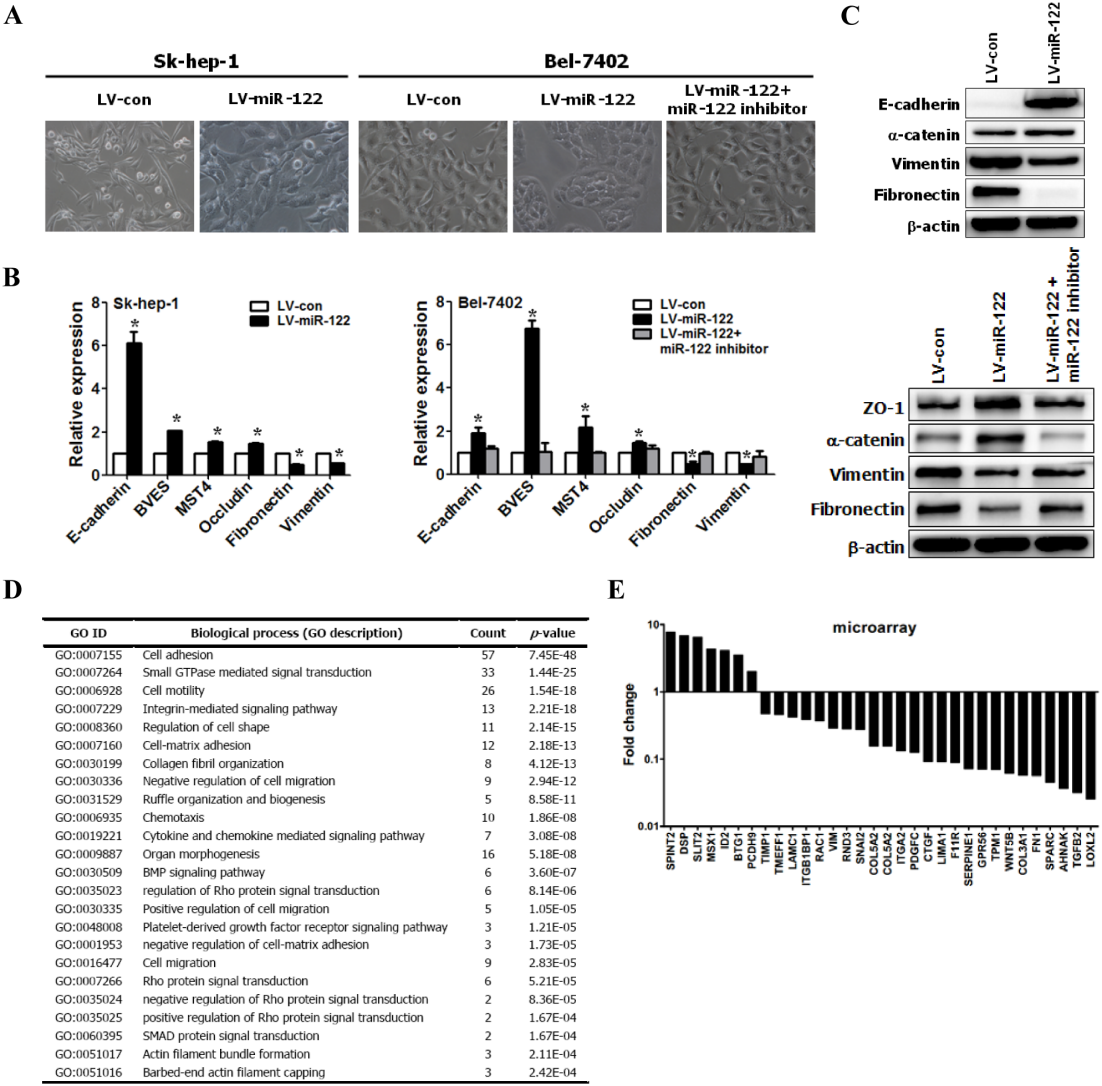
miR-122 induced epithelial-like phenotypes and MET-like cellular marker alterations in miR-122-expressing HCC cells. (**A**) Phase contrast images showing the morphology of Sk-hep-1 and Bel-7402 cells expressing either the control vector or miR-122 (Original magnification: ×200). (**B**) mRNA levels of the indicated genes in miR-122-expressing Sk-hep-1 and Bel-7402 cells based on qRT-PCR. (**C**) Western blot analysis of the indicated proteins in miR-122-expressing Sk-hep-1 and Bel-7402 cells. (**D**) Gene ontology (GO) analysis of the up- and down-regulated genes (associated with EMT, migration, and metastasis) from miR-122- and empty vector-expressing Sk-hep-1 cells. We identified and classified mRNAs showing a greater than 2-fold effect in expression with P values below 0.05 using GO categories. (**E**) Graph illustrating the fold-changes in the expression of EMT-, migration- and metastasis-related genes between miR-122- and empty vector-expressing Sk-hep-1 cells. miR-122-expressing Sk-hep-1 and control cells were analyzed using Affymetrix arrays. To examine the effects of the loss of miR-122 expression in liver cancer cells, miR-122 expression was down-regulated using a miR-122 inhibitor in miR-122-overexpressing Bel-7402 cells (Figures 1A, B, C).

The miR-122 transgene was over-expressed in Sk-hep-1 and Bel-7402 cells (Figure S1 in File S1), and these cells underwent significant epithelial-like morphological changes (Figure 1A), suggesting that miR-122 induced MET. Therefore, we characterized the MET at the molecular level by measuring the expression levels of a number of proteins markers. The results of the qRT-PCR analysis showed increased expression of epithelial markers, such as cell adhesion proteins (E-cadherin and BVES) and tight junction protein (occludin), and reduced expression of mesenchymal markers (vimentin and fibronectin) in miR-122-expressing Sk-hep-1 and Bel-7402 cells (Figure 1B). Western blot analysis confirmed the increased expression of E-cadherin and α-catenin in miR-122-expressing Sk-hep-1 cells and the increased expression of ZO-1 (zona occludens 1) and α-catenin in miR-122-expressing Bel-7402 cells. A concomitant decrease in the expression of mesenchymal markers (vimentin and fibronectin) was observed in miR-122-expressing Sk-hep-1 and Bel-7402 cells (Figure 1C). Furthermore, DNA microarray results also highlighted the occurrence of MET based on the expression changes of a large number of proteins (Figures 1D, E and Table S4 in File S1). A miR-122 inhibitor was transiently transfected into miR-122-expressing Bel-7402 cells to confirm that miR-122 induced MET. We found that the inhibition of miR-122 reversed the MET phenotypes observed in miR-122-expressing cells (Figures 1A, B, C).

**Figure 2:**
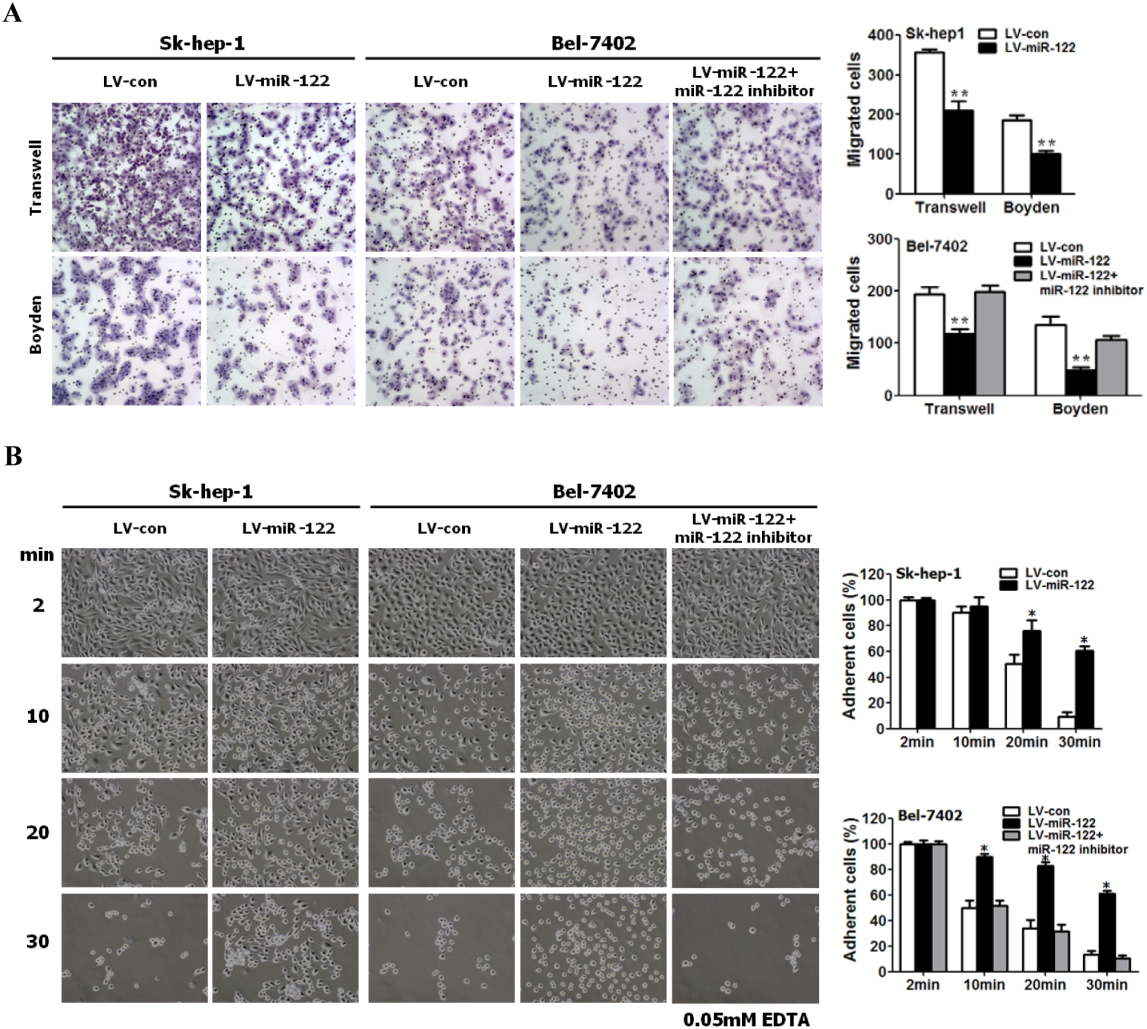
Suppressed migration and invasion and enhanced adhesion of miR-122-expressing HCC cells undergoing epithelial-like morphological changes. (**A**) miR-122-expressing Sk-hep-1 and Bel-7402 cells exhibited less motility and invasion than control cells. The motility and invasion of miR-122-expressing Sk-hep-1 and Bel-7402 cells were analyzed with an *in vitro* migration assay using a transwell chamber and an *in vitro* invasion assay using a Matrigel-coated Boyden chamber, respectively. The migrated cells were plotted as the average number of cells per field of view from 3 different experiments, as described in the Materials and methods section. (**B**) miR-122-expressing Sk-hep-1 and Bel-7402 cells exhibited increased cell adhesion. Cell adhesion assays were performed as described in the Materials and methods. The number of adherent cells was counted to determine the degree of cell adhesion. miR-122-expressing cells exhibited higher rates of adhesion than the control cells. Furthermore, to examine the influences of the loss of miR-122 expression on the motility, invasion and adhesion of liver cancer cells, miR-122 expression was inhibited using a miR-122 inhibitor on miR-122-expressing Bel-7402 cells (Figures 2A, B).

The loss of cell adhesion, inhibition of E-cadherin expression, and increase in cell motility are characteristics of EMT, the reverse process of MET [34]. As shown in Figure 2A, miR-122-expressing Sk-hep-1 and Bel-7402 cells exhibited epithelial-like phenotypes with significantly reduced cell migration and invasion and significantly enhanced cell adherence (Figure 2B). The inhibition of miR-122 reversed these epithelial-like phenotypes induced by miR-122 over-expression.

### The ectopic expression of miR-122 inactivated the RhoA/Rock pathway and impaired cytoskeletal events

**Figure 3:**
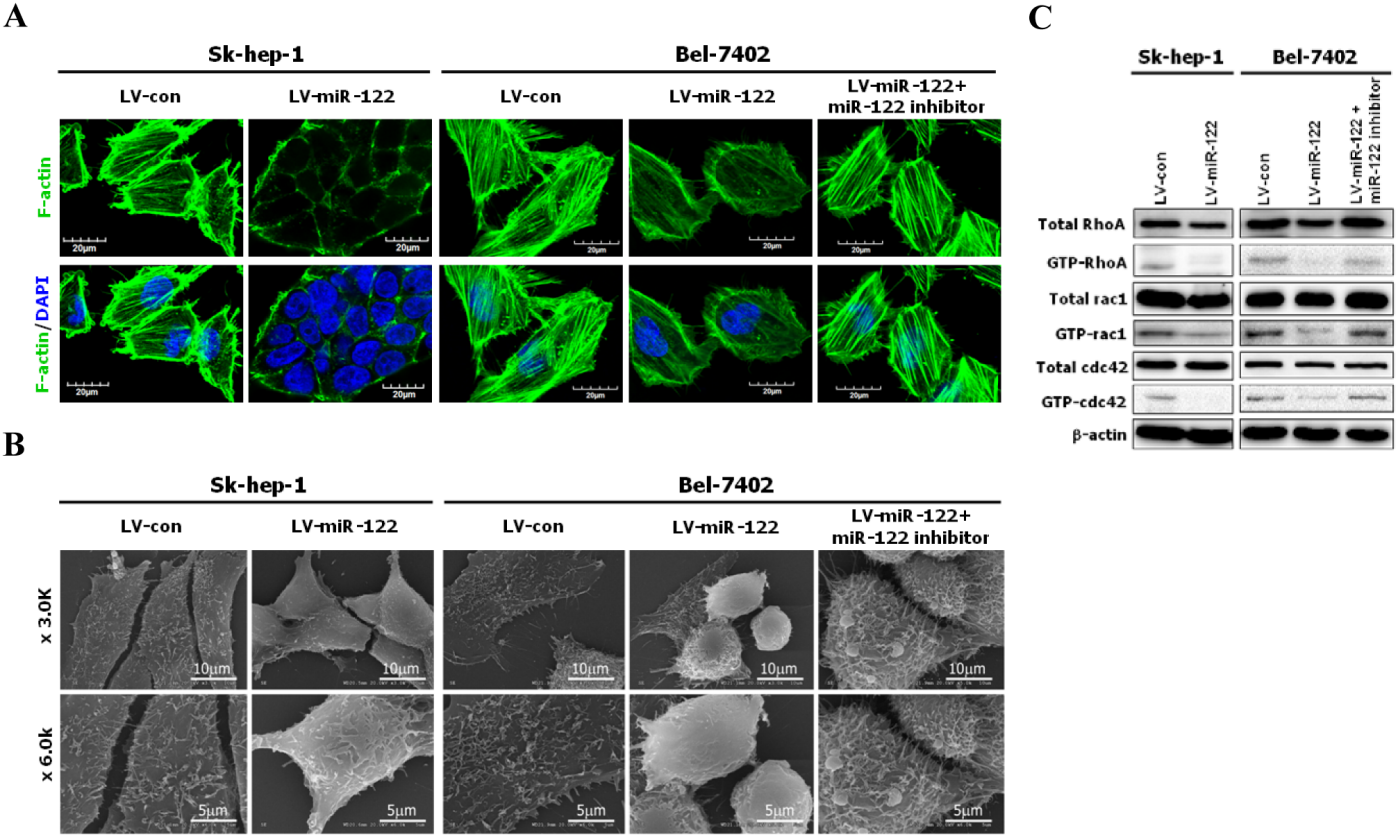
Ectopic expression of miR-122 in HCC cells suppressed cytoskeletal events and inactivated RhoA/Rock pathway. (**A**) Ectopic expression of miR-122 in Sk-hep-1 and Bel-7402 cells disrupted F-actin stress fiber networks. The formation of stress fibers (polymerized actin) and actin filaments was demonstrated by phalloidin staining (green). Empty vector-expressing Sk-hep-1 and Bel-7402 cells showed numerous stress fibers, whereas miR-122-expressing Sk-hep-1 and Bel-7402 cells showed a loss of stress fibers. (**B**) Ectopic expression of miR-122 in Sk-hep-1 and Bel-7402 cells suppressed the formation of filopodia and lamellipodia. (**C**) Western blot analysis comparing the total and active forms of Rho-GTPases, including RhoA, Rac1, and Cdc42, between empty vector- and miR-122-expressing cells. GTP-bound (active) forms of RhoA, Rac1 and Cdc42 were pulled down and detected by Western blot analysis using the corresponding antibodies. Furthermore, to explore the influences of the loss of miR-122 expression on cytoskeleton, filopodia, and lamellipodia formation and the RhoA/Rock pathway, the miR-122 expression in Bel-7402 cells was inhibited using a miR-122 inhibitor (Figures 3A, B, C).

The RhoA/Rock pathway is involved in EMT and MET by regulating cytoskeletal events such as the formation of stress fiber bundling arrays [29]–[31]. Therefore, we characterized the status of stress fiber formation and actin polymerization in miR-122-expressing cells. Using phalloidin staining, we observed that stress fiber formation was suppressed in miR-122-expressing cells, but not in empty vector-expressing cells. In addition, cytoskeletal disruption in miR-122-expressing cells could be restored by a miR-122 inhibitor (Figure 3A).

Filopodia and lamellipodia, dynamic structures on cell membrane, are involved in cell motility and cancer cell invasion and metastasis. The formation of filopodia and lamellipodia requires actin polymerization. Under a scanning electron microscope, a significant reduction or loss of filopodia and lamellipodia (cell protrusion) was observed on the membrane of miR-122-expressing cells, whereas miR-122 inhibition restored the formation of filopodia and lamellipodia in miR-122-expressing Bel-7402 cells (Figure 3B).

Previous studies have reported that RhoA induced the formation of stress fibers and Rac1 and Cdc42 stimulated the formation of lamellipodium and filopodia [29]–[31]. Pull-down assays were employed to investigate the active forms of Rho-GTPases. As shown in Figure 3C, higher levels of the active forms of RhoA, Rac1 and Cdc42 were observed in empty vector-expressing cells compared with miR-122-expressing cells. In addition, the RhoA/Rock pathway could be reactivated by the inhibition of miR-122 in Bel-7402 cells.

### miR-122 induced MET and inhibited the migration and invasion of HCC cells by targeting RhoA

**Figure 4:**
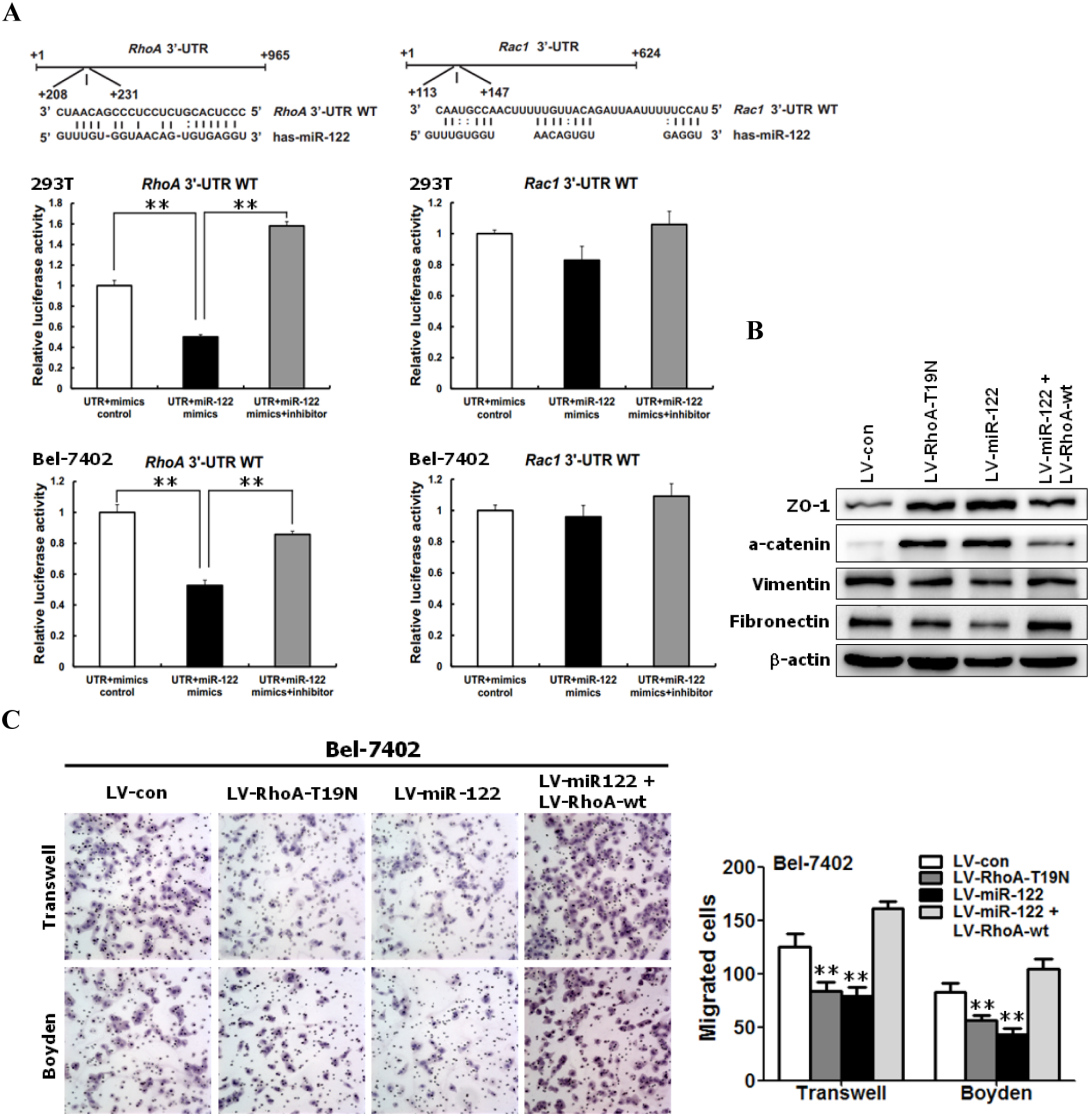
miR-122 induced MET, and suppressed cell migration and invasion by down-regulating RhoA. (**A**) RhoA is a target gene of miR-122. The luciferase reporter assay was performed using Bel-7402 and 293T cells as described in the Materials and methods section. (**B**) miR-122 triggered MET-like cellular marker alterations through the down-regulation of RhoA. Western blot analysis comparing the expression of epithelial markers (ZO-1 and α-catenin) and mesenchymal markers (vimentin and fibronectin) in Bel-7402 cells expressing the control vector, miR-122, miR-122/RhoA-wt, and RhoA-T19N. (**C**) miR-122 inhibited cell migration and invasion *in vitro* through RhoA targeting. The effects of miR-122, miR-122/RhoA-wt, and RhoA-T19N on cell migration and invasion were evaluated using transwell and Matrigel-coated Boyden chambers, respectively.

First, RhoA and Rac1 were identified as two potential targets of miR-122 by searching the miRNA databases microRNA.org, RNAhybrid, and miRWalk. The 3′-UTR of RhoA and Rac1 mRNAs contains a complementary site for the seed region of miR-122 (Figure 4A). Notably, previous studies have shown that RhoA and Rac1 played central roles in cell motility by regulating the cytoskeletal signaling pathway [29]–[31].

To determine whether miR-122 regulates RhoA and Rac1, we measured changes in RhoA and Rac1 expressions in HCC cells after the over-expression or inhibition of miR-122. The results of Western blotting revealed that total and active forms of both RhoA and Rac1 were dramatically reduced after the ectopic expression of miR-122 in Sk-hep-1 and Bel-7402 cells (Figure 3C). In addition, the knockdown of miR-122 using a miR-122 inhibitor increased the levels of total and active forms of RhoA and Rac1 (Figure 3C). Therefore, miR-122 negatively regulates RhoA and Rac1 in HCC cells.

We performed a luciferase reporter assay to determine whether miR-122 directly targets the 3′-UTRs of RhoA and Rac1. Transient transfection with wild-type RhoA-luc reporter and miR-122 mimics into 293T and Bel-7402 cells significantly reduced luciferase activity compared with mimics control (Figure 4A), whereas transient transfection with wild-type Rac1-luc reporter and miR-122 mimics could not significantly reduce the luciferase activity (Figure 4A). The results of qRT-PCR showed that RhoA mRNA levels were reduced in miR-122-expressing Sk-hep-1 and Bel-7402 cells, and the knockdown of miR-122 using a miR-122 inhibitor restored the levels of RhoA mRNA (Figure S2 in File S1). Therefore, these results suggest that RhoA, rather than Rac1, is a direct target of miR-122.

To elucidate whether the miR-122-induced MET and inhibition of cell motility and invasion are mediated through RhoA in HCC cells, we performed gain- and loss-of-function experiments. First we inhibited the function of wild-type RhoA using a dominant-negative mutant of RhoA (RhoA-T19N) to examine of the role RhoA in EMT, and migration and invasion of HCC cells. As shown in Figure 4, similar to miR-122 over-expression, the loss of RhoA function through RhoA T19N increased the expression of epithelial markers (ZO-1 and α-catenin) (Figure 4B), reduced the expression of mesenchymal markers (fibronectin and vimentin) (Figure 4B), and suppressed the motility and invasion of Bel-7402 cells (Figure 4C).

Subsequently, we evaluated whether the ectopic expression of RhoA reverses the miR-122-induced MET and rescues the miR-122-mediated suppression of migration and invasion of Bel-7402 cells. As expected, RhoA over-expression reversed the MET phenotypes (Figure 4B) and abrogated the suppressed motility and invasion (Figure 4C) induced by miR-122 in Bel-7402 cells.

Taken together, these results suggest that RhoA is the target of miR-122 in the regulation of EMT, motility and invasion of HCC cells.

### miR-122 was regulated by the transcriptional factor HNF4α to induce MET and inhibit the motility and invasion of HCC cells

Previous studies have demonstrated that miR-122 was positively associated with the expression of the liver-enriched transcription factors, such as HNF1A, HNF3A, HNF3B, HNF4A (HNF4α), HNF4G, and HNF6 [17], essential for hepatocyte differentiation. For example, it has been reported that HNF4α bound to the miR-122 promoter region to regulate miR-122 expression in HCC cells in mouse livers [35]. Other studies have also reported that HNF4α inhibited cell proliferation, EMT, invasion and metastasis of HCC cells [17], [36]–[39]. Therefore, we aimed to determine whether HNF4α directly regulates miR-122-induced MET and inhibition of migration and invasion of HCC cells.

**Figure 5:**
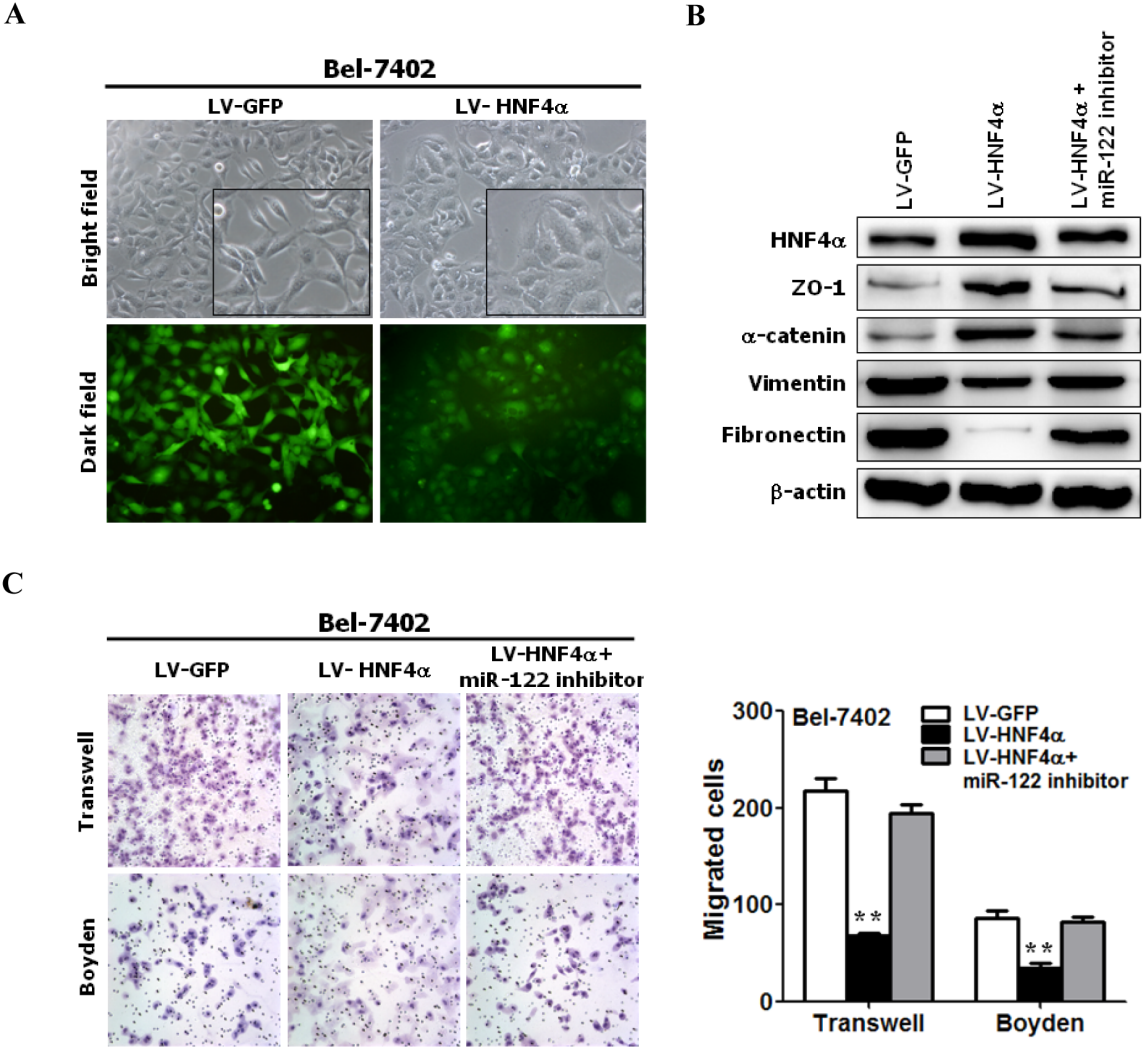
HNF4α over-expression induced MET-like cellular marker alterations and suppressed cell motility and invasion by up-regulating miR-122. (**A**) The over-expression of HNF4α induced an epithelial-like phenotype. Phage contrast images of the morphology of Bel-7402 cells expressing either the control vector or HNF4α are shown (original magnification: ×200). (**B**) The ectopic expression of HNF4α triggered MET-like cellular marker alterations through the up-regulation of miR-122. The cell extracts from Bel-7402 cells carrying the control vector, HNF4α or HNF4α and miR-122 inhibitor were analyzed through Western blotting with antibodies against the indicated proteins. (**C**) HNF4α over-expression suppressed cell motility and invasion in Bel-7402 cells via up-regulation of miR-122. The motility and invasion of Bel-7402 cells carrying the control vector, HNF4α or HNF4α and miR-122 inhibitor were analyzed using transwell and Matrigel-coated Boyden chambers, respectively. The migrated cells were plotted as the average number of cells per field of view from 3 different experiments, as described in the Materials and methods section.

We found that the ectopic expression of HNF4α (Figure S3 in File S1) increased miR-122 expression in Bel-7402 cells (Figure S4 in File S1), and HNF4α-expressing cells underwent significant epithelial-like morphological changes (Figure 5A). Western blot analysis validated the increase in ZO-1 and α-catenin expressions and the reduction of vimentin and fibronectin expressions in HNF4α-expressing Bel-7402 cells (Figure 5B). Therefore, HNF4α over-expression induced epithelial-like phenotypes and MET-like cellular marker alterations in HCC cells, similar to the results of restoring miR-122 expression in HCC cells (Figure 1C).

We also studied the changes in the motility and invasion of HNF4α-expressing cells. Similar to miR-122 over-expression (Figure 2A), HNF4α-expressing Bel-7402 cells exhibited significantly decreased mobility and invasion (Figure 5C). In addition, the repression of endogenous miR-122 expression by a miR-122 inhibitor reversed HNF4α-induced MET-like cellular marker alterations (Figure 5B) and abolished HNF4α-suppressed motility and invasion of Bel-7402 cells (Figure 5C).

Taken together, these results suggest that HNF4α is involved in the EMT, motility and invasion of HCC cells by regulating miR-122.

## Discussion

The majority of cancer deaths reflect tumor metastasis, thus the prevention of metastasis has received much attention [29]–[31]. EMT is an early event that is critical for cancer invasion and metastasis. To facilitate cell motility and invasion, epithelial cells lose adherence, tight junction proteins, polarity and cell-cell contact and undergo remarkable cytoskeleton remodeling [29]–[31]. Thus, understanding the molecular mechanisms of EMT will help elucidate the events involved in the metastatic cascade and might lead to the development of novel therapeutics for treating metastatic cancers.

Previous studies have shown that miR-122 negatively regulated invasion and metastasis in HCC [16], [17], [21], [23], [25], [27], [28]. However, the underlying mechanisms are not well understood. The results of the present study demonstrated that the reintroduction of miR-122 into Sk-hep-1 and Bel-7402 cells induced epithelial-like morphological conversion, accompanied by the increased expression of epithelial markers (E-cadherin, ZO-1 and α-catenin), decreased expression of mesenchymal markers (vimentin and fibronectin), increased cell adhesion, cytoskeleton depolymerization, RhoA/Rock pathway inactivation, and decreased cell motility and invasion. In addition, the knockdown of miR-122 reversed these phenotypes. These results are consistent with previous studies, showing that epithelial-like phenotypes resulted from the over-expression of miR-122 in hepG2 [16], [25] and Malhlavu cells [28], accompanied by the up-regulation of E-cadherin [25] and down-regulation of vimentin [16], [28]. Furthermore, the DNA microarray results highlighted the corresponding molecular features of miR-122-expressing Sk-hep-1 cells (Figures 1D, E and Table S4 in File S1), strongly supporting the aforementioned biological transformations. Collectively, the results of the present study and those obtained in other studies [16], [25], [28] fully support that the ectopic expression of miR-122 in liver cancer cells triggers MET that may contribute to the inhibition of motility and invasion of cancer cells.

As a cell moves in a designated direction, the cooperation between continuous actin polymerization and depolymerization facilitates the protrusion of the cell at the anterior front end [40]. In addition, the cell undergoes consecutive actomyosin contractions and separates from the posterior end, resulting in directional cell movement [40]. Moreover, the formation of the stress fibers, filopodia and lamellipodia also requires actin polymerization [40]. The loss of these structural features indicates an inability to engage in actin polymerization, resulting in reduced cell motility and invasion [40]. The results of the present study demonstrated that the exogenous expression of miR-122 in Sk-hep-1 and Bel-7402 cells substantially inhibited the RhoA/Rock pathway and suppressed the formation of stress fibers, filopodia and lamellipodia, whereas the inhibition of miR-122 restored the formation of stress fibers, filopodia and lamellipodia. These observations suggest that miR-122 suppressed HCC cell invasion and metastasis by regulating the formation of stress fibers, filopodia and lamellipodia.

Subsequently, we identified RhoA as a potential functional target of miR-122. It has been reported that RhoA is up-regulated in several types of human cancers, including HCC, and the over-expression of this protein has been positively associated with tumor metastasis and/or poor prognosis [41]–[49]. This idea is consistent with the findings obtained in the present study and other studies showing that the loss or down-regulation of miR-122 led to tumor metastasis and more aggressive and/or poor prognostic HCC phenotypes [8], [16]–[19], [21], [23], [25], [27], [28], [41]–[49]. A single miRNA can potentially regulate multiple genes, the results of the present study suggest that RhoA is a bona fide target of miR-122.

Notably, RhoA is a key component of the RhoA/Rock pathway, a well-characterized regulator of cytoskeletal organization and a positive regulator of EMT and the motility, invasion and metastasis of cancer cells, including HCC cells [29]–[31]. Thus, miR-122 induces MET and negatively modulates cell motility and invasion through the direct inhibition of RhoA in HCC cells.

**Figure 6:**
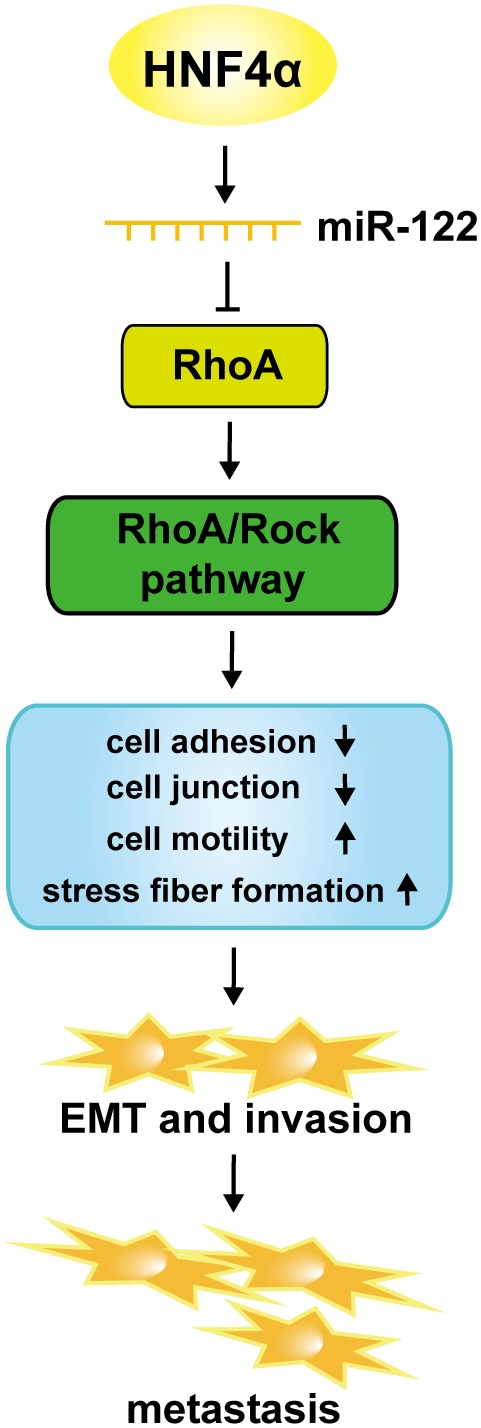
A proposed model for the HNF4α/miR-122/RhoA axis in regulating EMT, and the invasion and metastasis of HCC cells.

In the present study, we observed that loss of RhoA function through RhoA-T19N largely mimicked miR-122-induced MET, and the inhibition of migration and invasion of tumor cells, whereas RhoA over-expression in miR-122-expressing Bel-7402 cells reversed miR-122-induced MET and abrogated the miR-122-mediated suppression of motility and invasion of HCC cells. These findings suggest that miR-122 induced MET and inhibited the migration and invasion of HCC cells by targeting RhoA. To play multiple roles in diverse biological processes, miRNAs are mainly regulated by upstream transcriptional factors [50]. Previous studies have demonstrated that miR-122 was strongly associated with HNF4α expression in HCC tissues. Moreover, it has been reported that miR-122 is a direct target of HNF4α [35]. Similar to miR-122, the loss of HNF4α expression in HCC has been associated with a gain of invasive and metastatic properties [17]. However, whether HNF4α directly targets miR-122 to regulate miR-122-induced MET and inhibition of migration and invasion of HCC cells has not yet been reported. The results of the present study demonstrated that the ectopic expression of HNF4α induced MET to inhibit HCC cell motility and invasion through the up-regulation of miR-122 expression. Both HNF4α [17], [36], [37] and miR-122 [16], [17], [21], [23], [25], [27], [28] act as tumor suppressors and negative regulators of HCC invasion and metastasis, whereas RhoA positively regulates HCC invasion and metastasis [41], [43], [49]. Furthermore, miR-122 overexpression could promote hepatic differentiation and maturation of mouse embryonic stem cells (ESC)-derived cells by regulating the balance between EMT and MET at least in part through a miR-122/FoxA1/HNF-4a positive feedback loop [51]. Based on the results of the present study and previous studies [16], [17], [21], [23], [25], [27], [28], [35]–[37], [41], [43], [49], we propose that HNF4α induces miR-122 expression, which subsequently suppresses RhoA, thereby inactivating the RhoA/Rock pathway, enhancing cell adhesion and cell junction, impairing cytoskeletal events, and decreasing cell motility (Figure 6). In summary, these results illustrate that the HNF4α/miR-122/RhoA axis negatively regulates EMT and the invasion and metastasis of HCC cells (Figure 6).

In conclusion, these findings demonstrate, for the first time, that miR-122 that is regulated by the upstream transcriptional factor HNF4α triggers MET to suppress HCC cell motility and invasion by targeting RhoA. Thus, miR-122 might be a promising therapeutic target for the inhibition of invasion and metastasis of HCC.

## Supporting Information

File S1 **Figures S1–S4 and Tables S1–S4.** Figure S1. qRT-PCR analysis of miR-122 expression in Sk-hep-1 and Bel-7402 cells with miR-122 over-expression or miR-122 inhibitor. Figure S2. qRT-PCR analysis of RhoA expression in Sk-hep-1 and Bel-7402 cells with miR-122 over-expression or miR-122 inhibitor. Figure S3. qRT-PCR analysis of HNF4α expression in Bel-7402 cells with HNF4α over-expression. Figure S4. qRT-PCR analysis of the miR-122 expression in Bel-7402 cells carrying the HNF4α transgene or the miR-122 inhibitor. Table S1. Primers for qRT-PCR analysis of human miR-122. Table S2. Primers for qRT-PCR analysis of MET-related gene. Table S3. List of antibodies and suppliers used for immunoblotting. Table S4. Gene ontology (GO) of differentially expressed genes (related with EMT, migration and metastasis) from miR-122-expressing Sk-hep-1 cells to vector-expressing Sk-hep-1 cells.
(ZIP)
