# Supplementary material for: MicroRNA-122 Triggers Mesenchymal-Epithelial Transition and Suppresses Hepatocellular Carcinoma Cell Motility and Invasion by Targeting RhoA
Source: PLoS One. 2014 Jul 3;9(7):e101330. doi: 10.1371/journal.pone.0101330 (PMC4081555; doi:10.1371/journal.pone.0101330)
Supplement: File S1 — Figures S1–S4 and Tables S1–S4. Figure S1. qRT-PCR analysis of miR-122 expression in Sk-hep-1 and Bel-7402 cells with miR-122 over-expression or miR-122 inhibitor. Figure S2. qRT-PCR analysis of RhoA expression in Sk-hep-1 and Bel-7402 cells with miR-122 over-expression or miR-122 inhibitor. Figure S3. qRT-PCR analysis of HNF4α expression in Bel-7402 cells with HNF4α over-expression. Figure S4. qRT-PCR analysis of the miR-122 expression in Bel-7402 cells carrying the HNF4α transgene or the miR-122 inhibitor. Table S1. Primers for qRT-PCR analysis of human miR-122. Table S2. Primers for qRT-PCR analysis of MET-related gene. Table S3. List of antibodies and suppliers used for immunoblotting. Table S4. Gene ontology (GO) of differentially expressed genes (related with EMT, migration and metastasis) from miR-122-expressing Sk-hep-1 cells to vector-expressing Sk-hep-1 cells. (ZIP) [file pone.0101330.s001.zip › Table S4.doc]

**Table S4. Gene ontology (GO) of differentially expressed genes (related with EMT, migration and metastasis) from miR-122-expressing Sk-hep-1 cells to vector-expressing Sk-hep-1 cells**

**GO terms representing biological process**

| **GO ID** | **GO description** | **Count** | **Genes** | ***p*-value** |
| --- | --- | --- | --- | --- |
| GO:0007155 | Cell adhesion | 57 | DDR1;CYR61;RPSA;PKP3;GPR56;LAMB3;PXN;MSLN;CFDP1;IGFBP7;CASK;CTNNA1;ERBB2IP;FN1;ITGB3BP;LOXL2;VCL;HES1;CDH2;ALCAM;ITGB5;PCDH7;LGALS3BP;CADM1;TNFRSF12A;PDPN;APP;THBS1;JUB;BCAR1;CTGF;MLLT4;  CD9;ARF6;ARVCF;CXADR;PNN;HEPACAM;DST;DCBLD2;NRP1;CDH18;SEMA4D;ITGB2;ITGA2;F11R;ITGB1BP1;ITGA6;PTPRM;FAT1;BOC;  RAC1;EMR1;LAMC1;PCDH9;RND3;LAMB1 | 7.45E-48 |
| GO:0007264 | Small GTPase mediated signal transduction | 33 | RAC2;ARL4A;RABL4;RP1BL_HUMAN;RAP1B;  RIN2;RAB2A;RAB3B;BCAR3;RAB24;RAB22A;  PARF;RAB10;RHOD;ARF6;ARHGAP1;RRAD;  YWHAQ;HMOX1;RAB9A;RAB8A;RHOC;RAC1;  RAB32;RAB8B;RAB21;RAP2B;ARFIP2;RND3;  RAC3;ARL4C;RAB34;ARL5B | 1.44E-25 |
| GO:0006928 | Cell motility | 26 | ACTG1;TPM1;STAT3;TUBB2C;ARHGDIB;TUBB;PLAUR;ACTB;VIM;TPM4;VCL;ARPC1B;CAPZA2;  ARPC2;TNFRSF12A;MYH9;CD9;ACTR3;ARF6;  TPM3;DSTN;ACTR2;RAC1;SPINT2;ARFIP2;  ANXA1 | 1.54E-18 |
| GO:0007229 | Integrin-mediated signaling pathway | 13 | ERBB2IP;ITGB5;COL3A1;MYH9;BCAR1;CTGF;  DST;CCM2;ITGA2;ITGA6;ITGB1;PLP1;ILK | 2.21E-18 |
| GO:0008360 | Regulation of cell shape | 11 | CFDP1;FN1;CDC42EP3;PALM2;RASA1;DLC1;  EZR;MYH10;MYH9;PDPN;ALDOA | 2.14E-15 |
| GO:0007160 | Cell-matrix adhesion | 12 | SGCE;PXN;CD44;ITGB5;COL3A1;CDK5;CTGF;  ITGA2;ITGB1BP1;ITGB1;ILK;RAPH1 | 2.18E-13 |
| GO:0030199 | Collagen fibril organization | 8 | NF1;LOX;FOXC1;COL5A2;ANXA2;COL3A1;  SERPINH1;TGFB2 | 4.12E-13 |
| GO:0030336 | Negative regulation of cell migration | 9 | TPM1;CITED2;NF1;VCL;THY1;CLIC4;DLC1;  RRAS;PTEN | 2.94E-12 |
| GO:0031529 | Ruffle organization and biogenesis | 5 | TPM1;LIMA1;ARF6;RAC1;ARFIP2 | 8.58E-11 |
| GO:0006935 | Chemotaxis | 10 | RAC2;CYR61;CMTM3;PLAUR;PLAU;TYMP;  SLIT2;PLP2;FGF2;CMTM8 | 1.86E-08 |
| GO:0019221 | Cytokine and chemokine mediated signaling pathway | 7 | KLF6;IL1B;SOCS5;PLP2;IL6ST;JAK1;TNFRSF1A | 3.08E-08 |
| GO:0009887 | Organ morphogenesis | 16 | NOTCH2;CSRP2;PAX6;SMARCC1;HEY1;GYPC;  ETV4;TLE2;PHLDA2;AES;FHL1;NRP1;FGF2;  PDGFC;ITGA2;GSK3B | 5.18E-08 |
| GO:0030509 | BMP signaling pathway | 6 | SMURF2;FSTL1;CHRDL1;MSX1;ID1;SMAD4 | 3.60E-07 |
| GO:0035023 | regulation of Rho protein signal transduction | 6 | DNMBP;TRIO;AKAP13;ITSN1;ARHGEF5;SOLO | 8.14E-06 |
| GO:0030335 | Positive regulation of cell migration | 5 | TRIP6;EGFR;PDPN;BCAR1;LAMB1 | 1.05E-05 |
| GO:0048008 | Platelet-derived growth factor receptor signaling pathway | 3 | BCAR1;PTEN;PDGFC | 1.21E-05 |
| GO:0001953 | negative regulation of cell-matrix adhesion | 3 | BCL6;RASA1;CDKN2A | 1.73E-05 |
| GO:0016477 | Cell migration | 9 | FN1;PSG2;CCDC88A;CTGF;TNS3;BTG1;CDH2;  ITGB1BP1;LAMC1 | 2.83E-05 |
| GO:0007266 | Rho protein signal transduction | 6 | ARHGDIB;TAX1BP3;ARHGAP29;RHOD;  ARHGAP1;ARHGAP4 | 5.21E-05 |
| GO:0035024 | negative regulation of Rho protein signal transduction | 2 | BCL6;DLC1 | 8.36E-05 |
| GO:0035025 | positive regulation of Rho protein signal transduction | 2 | LPAR1;RAC1 | 1.67E-04 |
| GO:0060395 | SMAD protein signal transduction | 2 | CITED1;SMAD4 | 1.67E-04 |
| GO:0051017 | Actin filament bundle formation | 3 | ACTN4;LIMA1;EZR | 2.11E-04 |
| GO:0051016 | Barbed-end actin filament capping | 3 | SVIL;CAPZA2;CAPG | 2.42E-04 |

**Supporting Results**

To identify genes under the control of miR-122, we compared the changes in the mRNA expression profile between miR-122- and empty vector-expressing Sk-hep-1 cells using microarrays. The results demonstrated that 1401 genes were significantly down-regulated and 868 genes were significantly up-regulated in miR-122-expressing Sk-hep-1 cells compared with controls (data not shown). The up- and down-regulated genes derived from microarray data were submitted to the Database for Annotation, Visualization and Integrated Discovery (DAVID) (http://david.abcc.ncifcrf.gov/home.jsp) for gene ontology (GO) annotation. Figure 1D and Table S4 list the GO terms representing biological processes associated with EMT, migration and metastasis. The GO analysis of the 2269 genes displaying significant changes in expression in miR-122-expressing Sk-hep-1 cells illustrated a significant enrichment for 257 genes with functions typically associated with EMT, migration. and metastasis [e.g., significant over-representation of genes involved in cell adhesion and cell-matrix adhesion, cell migration and motility, Rho protein signal transduction, regulation of cell shape, collagen fibril organization, ruffle organization and biogenesis, chemotaxis, BMP signaling pathway, integrin-mediated signaling pathway, cytokine and chemokine mediated signaling pathway, and small GTPase-mediated signal transduction] (Figure 1D and Table S4). Some selected EMT, migration and metastasis genes showing a fold-change of more than 2 are shown in Figure 1E.
