# Supplementary material for: MicroRNA-122 Triggers Mesenchymal-Epithelial Transition and Suppresses Hepatocellular Carcinoma Cell Motility and Invasion by Targeting RhoA
Source: PLoS One. 2014 Jul 3;9(7):e101330. doi: 10.1371/journal.pone.0101330 (PMC4081555; doi:10.1371/journal.pone.0101330)
Supplement: File S1 — Figures S1–S4 and Tables S1–S4. Figure S1. qRT-PCR analysis of miR-122 expression in Sk-hep-1 and Bel-7402 cells with miR-122 over-expression or miR-122 inhibitor. Figure S2. qRT-PCR analysis of RhoA expression in Sk-hep-1 and Bel-7402 cells with miR-122 over-expression or miR-122 inhibitor. Figure S3. qRT-PCR analysis of HNF4α expression in Bel-7402 cells with HNF4α over-expression. Figure S4. qRT-PCR analysis of the miR-122 expression in Bel-7402 cells carrying the HNF4α transgene or the miR-122 inhibitor. Table S1. Primers for qRT-PCR analysis of human miR-122. Table S2. Primers for qRT-PCR analysis of MET-related gene. Table S3. List of antibodies and suppliers used for immunoblotting. Table S4. Gene ontology (GO) of differentially expressed genes (related with EMT, migration and metastasis) from miR-122-expressing Sk-hep-1 cells to vector-expressing Sk-hep-1 cells. (ZIP) [file pone.0101330.s001.zip › Figure S1.doc]

**Figure S1**

******

******

**Figure S1. qRT-PCR analysis of miR-122 expression in Sk-hep-1 and Bel-7402 cells with miR-122 over-expression or miR-122 inhibitor.**
